# 

**Published:** 1903-10-31

**Authors:** 


### CONTENTS.

**Notification in Tuberculosis** - 75
**Materia Medica and the Medical Curriculum** - 76
**Annotations** - 77
**Notes and News** - 78
**Hospital Clinics and Medical Progress—**
The Ultimate Results obtained in the Open-air Treatment of Pulmonary Tuberculosis - 79
Tumours of the Bladder - 80
Urinary Casts - 81
Diffuse Gonocccus Infection - 81
Progress in Genito-Urinary Surgery - 82
Progress in State Medicine - 83
Progress in Bacteriology - 84
**New Appliances and Things Medical** - 85
**Hospital Administration—**
St. Bartholomew's Hospital and the Nation - 86
The Royal Waterloo Hospital for Women and Children - 89
The Mount Vernon Hospital for Consumption - 90
**Editor's Letter-Box** - 91
**The Student of Medicine** - 92

*NURSING SECTION.*

**Notes on News from the Nursing World—** Our Christmas Distribution—Queen Alexandra's Military Nursing Service—The Foreign Office and Nurses for the Balkans—American Superintendents in Conference—The Standard of Nursing in Switzerland—Royal United Hospital, Bath—Nurses Without Caps—The British Gynæcological Society—Nurses for Liners—The Nursing of Infants—Pontypool Hospital—Nurses and their Medical Superintendent—Granard Nursing Question—A District Association in the Wrong—The Nurses' Total Abstinence League—The Nurses' Hostel Company—Additional Nurses for West Ham Infirmary—District Nursing in Torquay—Progress at Crieff—New Home at Horsforth—Short Items - 63-66

**Some Practical Points in the Nursing of Diseases of the Skin** - 67

**The American Society of Training Schools for Nurses** - 68

**The Prevention of Bed-Sores** - 70

**Everybody's Opinion** - 71

**Presentations** - 71

**Travel Notes and Queries** - 72

**Appointments** - 72

**Echoes from the Outside World** - 73

**For Reading to the Sick** - 74

**Notes and Queries** - 74

